# Diagnostic Accuracy of Cerebrospinal Fluid Multiplex Polymerase Chain Reaction Panel Testing in Patients With Suspected Central Nervous System Infections: A Multi-Center Study in the United Arab Emirates

**DOI:** 10.7759/cureus.51906

**Published:** 2024-01-08

**Authors:** Yousra Ghoweba, Seyed Ali Safizadeh Shabestari, Zainab A Malik

**Affiliations:** 1 Pediatrics, Mohammed Bin Rashid University Of Medicine and Health Sciences, Dubai, ARE; 2 Medicine, Mohammed Bin Rashid University Of Medicine and Health Sciences, Dubai, ARE; 3 Pediatrics and Pediatric Infectious Diseases, Mohammed Bin Rashid University of Medicine and Health Sciences, Dubai, ARE; 4 Pediatrics and Pediatric Infectious Diseases, Genesis Healthcare, Dubai, ARE

**Keywords:** diagnostic, middle east, csf, mpcr, encephalitis, meningitis, cns

## Abstract

Background

Delays in diagnosis and treatment of central nervous system (CNS) infections can lead to significant morbidity and mortality among children and adults. Prior antibiotic treatment is a major hurdle to accurate diagnosis due to falsely negative cerebrospinal fluid (CSF) cultures in partially treated patients. Increasingly, molecular diagnostic methods using multiplex polymerase chain reaction (mPCR) testing on CSF samples are being utilized in clinical practice for timely and accurate diagnosis. However, there is no data regarding the diagnostic accuracy or clinical impact of CSF mPCR testing in the Middle East region. We sought to compare the diagnostic accuracy of an automated mPCR CSF panel with routine CSF culture, the current gold standard, in the United Arab Emirates (UAE).

Methods

This single-gated, multi-center, diagnostic accuracy study included patients from birth onwards who were admitted to any of the three participating hospitals with an initial diagnosis of meningitis or encephalitis, between January 2017 and March 2021, and had CSF samples collected for mPCR and culture. Sociodemographic, clinical, and molecular data were collected for all.

Results

A total of 353 CSF samples were collected from patients from 0-90 years old hospitalized for suspected CNS infection. Children constituted 51% of the study population, and males were slightly over-represented (55.2%). Pathogens were detected by mPCR in 78 (22%) CSF samples, of which 19 (24%) were bacteria and 59 (76%) were viruses. No fungal pathogens were detected. Enteroviruses were the most prevalent CNS pathogen among our cohort (40%), followed by *herpes simplex virus type 2* (HSV-2) (12.5%). Children constituted 69% of positive samples for enterovirus, while HSV-2 was exclusively detected among adults. Using CSF culture as the diagnostic gold standard, the mPCR panel demonstrated high specificity (100%) and sensitivity (96.3%) in diagnosing CNS infection among all age groups. mPCR testing demonstrated a high overall percentage of agreement (OPA) with CSF culture (98.9%). Patients with bacterial meningitis had a significantly longer hospitalization (p=0.004) and duration of antibiotic therapy (p=0.001) compared to those with viral meningitis. Three CSF samples were negative on mPCR testing but positive on culture. These pathogens included: methicillin-sensitive *Staphylococcus aureus*(MSSA)*, Bacillus cereus, *and* Mycobacterium Tuberculosis *(MTB). In addition, 13 patients had negative CSF cultures but tested positive on CSF mPCR. These pathogens included *Streptococcus pneumoniae *(seven patients), *Haemophilus influenzae* (three patients), *Streptococcus agalactiae* (two patients), and *Escherichia coli* (one patient). All discordant results were confirmed by reviewing the patient’s clinical presentation, CSF analysis, clinical course, and final diagnosis.

Conclusion

CSF mPCR panel is a highly sensitive and specific diagnostic tool for the diagnosis of CNS infections among all age groups in the UAE. Routine use of CSF mPCR panels can decrease healthcare costs by reducing the length of stay and can also aid antibiotic stewardship efforts by reducing antibiotic overuse in patients with viral CSF infections. CSF culture and mPCR complement each other by identifying CNS pathogens in patients with prior antibiotic exposure who would otherwise be missed if relying on CSF culture alone. However, concomitant CSF culture samples should be sent to avoid missing unusual CNS pathogens.

## Introduction

Central nervous system (CNS) infections present significant challenges in clinical diagnosis due to their diverse etiologies and high morbidity and mortality [1]. Prompt and accurate identification of the causative pathogens is crucial for appropriate patient management and initiation of targeted therapy [2-3]. Early treatment is necessary to avoid complications such as seizures, subdural effusions, hemiparesis, and long-term neurologic impairment [4-5].

Traditional diagnostic methods, such as culture and microscopy, have limitations in sensitivity, specificity, and turnaround time, necessitating the development of more advanced diagnostic tools when CNS infections are suspected. Due to lengthy turnaround times for cerebrospinal fluid (CSF) culture results, patients with strong clinical suspicion of CNS infection often receive empirical antimicrobial therapy to mitigate potential complications arising from delayed management. Antibiotic treatment initiated prior to CSF sample collection can impact the accuracy of CSF culture results, leading to false-negative cultures. This results in lengthy hospitalizations, unnecessary antimicrobial use, and an overall increase in healthcare costs. Molecular diagnostic methods like polymerase chain reaction (PCR) detection can address these disadvantages by providing timely and accurate results that are unaffected by prior antimicrobial use. This allows the identification of patients with partially treated CNS infections who would otherwise go undetected using conventional CSF culture [3]. 

In this study, we evaluated the use of a rapid diagnostic multiplex PCR (mPCR) on CSF samples and compared it with the current gold standard, CSF culture. Despite the growing interest among clinicians, there is insufficient data regarding the efficacy and clinical impact of CSF mPCR testing in managing patients with CNS infections. The aim of our study was to determine the real-world impact of CSF mPCR testing in improving the diagnosis of CNS infections in Dubai, United Arab Emirates (UAE).

## Materials and methods

Study design and setting 

This single-gated, multi-center, diagnostic accuracy study was conducted within a multidisciplinary, university-affiliated healthcare network that comprises three large tertiary-care hospitals and ten outpatient clinics in Dubai, UAE. All these facilities are served by a centralized microbiology laboratory, which processes CSF mPCR samples from all sites within the healthcare network. Our study population included all patients from birth onwards, admitted to any of the three affiliated hospitals with an initial diagnosis of CNS infection (meningitis or encephalitis) and with CSF samples sent for routine culture and mPCR between January 1, 2017 to March 31, 2021. Patients were diagnosed with CNS infection based on their clinical presentation including fever, neck pain, nausea, vomiting, lethargy, and/or neurologic deficit, along with clinical findings of positive Kernig and/or Brudzinski sign, laboratory findings of leukocytosis with elevated inflammatory markers. Some patients also had MRIs with meningeal enhancement that was consistent with a CNS infection. We excluded patients if the indication for lumbar puncture was non-infectious, i.e., idiopathic intracranial hypertension, CNS tumors, CNS demyelinating disease, or others. Duplicate specimens from the same patient were also excluded.

Lumbar punctures were performed under aseptic conditions and CSF samples for culture and mPCR were collected in sterile CSF transport tubes and transported to the centralized laboratory. Samples collected in the hospital attached to the centralized laboratory were dispatched immediately, while those collected at the two off-site hospitals were refrigerated and dispatched within one hour of collection, 24 hours a day, 365 days a year. Samples were tested using BioFire® FilmArray® meningitis/encephalitis multiplex PCR (BioFire Diagnostics, Salt Lake City, Utah, United States), which tests for six bacteria *(Escherichia coli (E. coli) gK1, Haemophilus influenzae (H. influenzae*)*, Listeria monocytogenes (L. monocytogenes*​​​​​​​)*, Neisseria meningitidis (N. meningitidis*​​​​​​​)*, Streptococcus agalactiae (S. agalactiae​​​​​​​*)*, Streptococcus pneumoniae (S. pneumoniae)*, seven viruses (*Cytomegalovirus *(CMV)*, Enterovirus *(EV)*, Herpes simplex virus 1 *(HSV-1)*, Herpes simplex virus 2 *(HSV-2)*, Human herpesvirus 6 *(HHV-6)*, Human parechovirus *(HPeV)*, Varicella zoster virus *(VZV)), and a yeast (*Cryptococcus neoformans/gattii*) commonly responsible for community-acquired CNS infections [6]. It has a high manufacturer-reported sensitivity (94.2%) and specificity (99.8%) [6]. This is a closed, fully automated system that extracts the nucleic acids and runs the PCR cycle. The FilmArray® software analyzes and interprets the assay results, generating a final report within an hour of sample processing. The test report is reviewed and authorized by the microbiology laboratory staff, before being released to the patient’s electronic medical records (EMR). The CSF culture can detect bacteria and fungi. The central microbiology laboratory is fully staffed and runs 24 hours a day, seven days a week. It is accredited by the College of American Pathologists (CAP) and the Joint Commission International (JCI) and holds the ISO-15189 certification.

Data was collected for patient-related clinical, laboratory, imaging, demographic, and epidemiological variables. Antimicrobial use prior to the collection of CSF samples, the total duration of antimicrobial use, and the length of hospitalization were recorded when available. Molecular data for pathogen identification and CSF culture results were also collected.

Data was analyzed using the IBM SPSS Statistics for Windows, Version 25, (Released 2017; IBM Corp., Armonk, New York, United States). Frequencies with proportions were reported for categorical variables and mean with standard deviations (SD) were reported for continuous variables. Association between categorical variables was tested by the chi-square and Fischer Exact tests when appropriate. Sensitivity, specificity, positive predictive value (PPV), and negative predictive value (NPV) of the mPCR assay were calculated. The overall percentage of agreement (OPA) between mPCR and CSF culture was calculated as ((TP+TN)/(TP+TN+FP+FN)) ×100%. The duration of the antimicrobial course (One-Tailed T-test) and hospitalization (Two-Tailed T-test) among children, adults, and elderly were analyzed and expressed as the median and interquartile range (IQR). Similarly, these analyses were performed for cases of viral and bacterial meningitis. A p-value of <0.05 was considered statistically significant for all analyses.

All patients or caregivers signed a consent form for lumbar puncture. Since mPCR testing of CSF samples was performed as part of the routine testing protocol during the provision of standard clinical care, additional patient consent was not required. This study was reviewed and approved by the Institutional Review Boards (IRB) of Mediclinic Middle East (IRB reference number MCME. CR.174.MCIT.2021) and Dubai Healthcare City Authority (IRB reference number CP7.1.02_F05).

## Results

**Figure 1:**
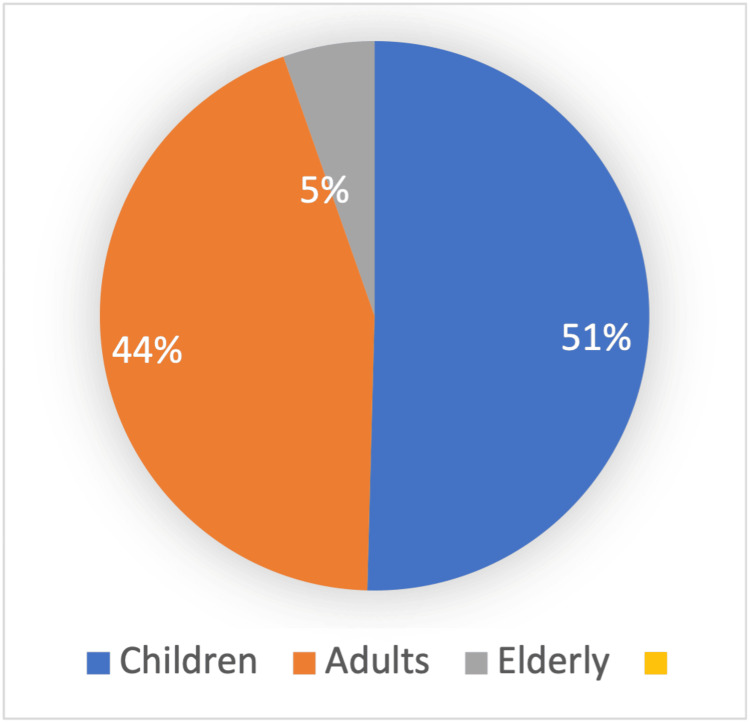
Age distribution of the study sample Children constituted 51% of the study sample, followed by 44% and 5% of adults and elderly, respectively.

Four hundred patients were hospitalized and underwent CSF sample collection during the study period. However, after excluding patients with non-infectious indications for lumbar puncture, 353 (88%) patients met the inclusion criteria and were included in the analysis. Children up to 18 years constituted 51% (180) of the study sample, while adults >18 to 60 years constituted 44% (155) and elderly >60 years constituted 5% (18) of the study population (Figure 1). Just over half the patients were male (55%) and 11% were UAE Nationals. One hundred and twenty-three patients (34.8%) received antibiotics prior to CSF testing.

**Figure 2:**
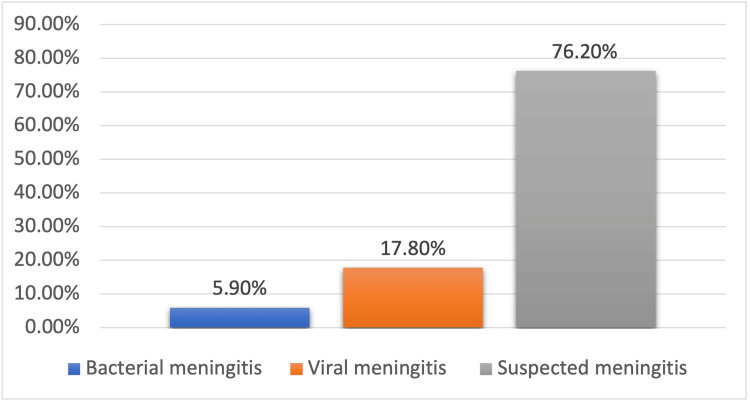
Clinical diagnosis of the study sample Viral meningitis was diagnosed in 17.80% of the study sample, while 5.09% were diagnosed with bacterial meningitis and 76.20% with suspected meningitis.

**Figure 3:**
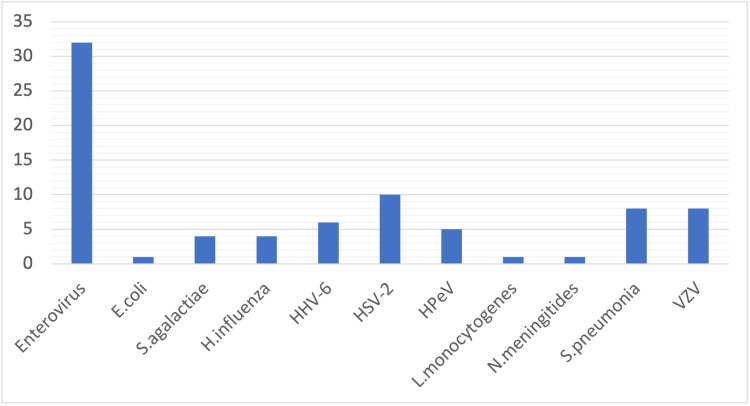
Number of detections of the cerebrospinal fluid polymerase chain reaction panel Out of the 78 positive PCR CSF samples, 61 viral pathogens were detected while 19 were bacterial pathogens. Two samples had two viral co-detections. No fungal pathogens were detected. Enteroviruses were the most prevalent CNS pathogen (32/80, 40.0%), followed by HSV-2 (10/80, 12.5%), *S. pneumonia* (8/80, 10.0%), and VZV (8/80, 10.0%). *E. coli*: *Escherichia coli; S. agalactiae*: *Streptococcus agalactiae; H. influenzae*: *Haemophilus influenzae; *HHV-6: *Human herpesvirus 6; *HSV-2:* Herpes simplex virus 2*; HPeV: *Human parechovirus; Listeria monocytogenes*: *L. monocytogenes; Neisseria meningitidis*: *N. meningitidis; S. pneumoniae*: *Streptococcus pneumoniae; *VZV: *Varicella zoster virus; *PCR: polymerase chain reaction; CSF: cerebrospinal fluid; CNS: central nervous system

**Figure 4:**
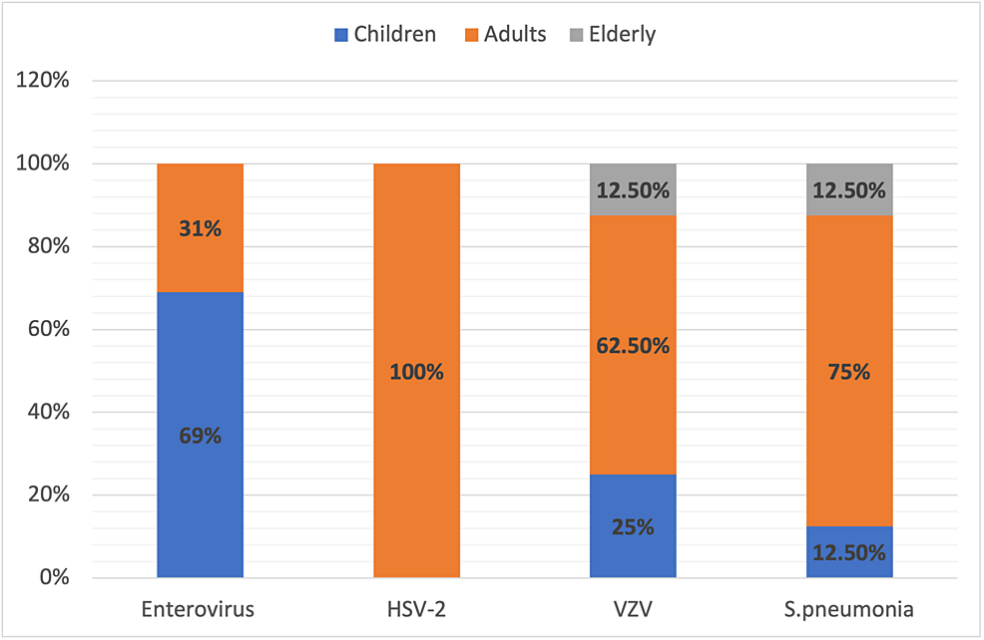
Age distribution of enterovirus, HSV-2, VZV, and S. pneumoniae Children constituted 69% of positive samples for enterovirus, while *HSV-2* was exclusively detected among adults. The majority of *VZV* and *S. pneumoniae*-positive samples were detected in adults. HSV-2: *Herpes simplex virus 2*; VZV: *Varicella zoster virus*; *S. pneumoniae*: *Streptococcus pneumoniae*

A total of 78 (22%) CSF samples were positive on mPCR assay. Two patients tested positive for two pathogens on mPCR samples; hence, a total of 80 pathogens were detected in 78 CSF samples. Among them, there were 61 viral pathogens and 19 bacterial pathogens. Twenty-one patients (5.9%) were diagnosed with bacterial meningitis, 63 (17.8%) with viral meningitis, and 269 (76.2%) had suspected meningitis (Figure 2). No patient had CSF mPCR positive for yeast. EVs were the most prevalent pathogen on CSF mPCR among all our patients (32/80, 40%), followed by HSV-2 (10/80, 12.5%), *S. pneumoniae* (8/80, 10%), and VZV (8/80, 10%) (Figure 3). CSF EV infections were more common among children (69%) than adults, while HSV-2 was exclusively detected among adults. The vast majority of VZV and *S. pneumoniae* mPCR positivity was noted among samples from adults, 62.5% and 75%, respectively. Among CSF samples from children, 25% were positive for VZV and 12.5% for *S. pneumoniae *(Figure 4).

Using CSF culture as the diagnostic gold standard, the sensitivity and specificity of the CSF mPCR panel were calculated. The mPCR panel demonstrated a sensitivity of 96.3% and a specificity of 100%, with an overall percentage agreement (OPA) between mPCR and CSF culture of 99.2% as seen in Table 1. There were three CSF samples that were positive on culture, but negative on mPCR testing. Pathogens identified in these samples were methicillin-sensitive *Staphylococcus aureus* (MSSA) in a neonate, *Bacillus cereus (B.cereus)* in a 31-rear-old, and *Mycobacterium tuberculosis (MTB)* in a 30-year-old patient. Antibiotics were administered prior to lumbar puncture for the patient with *MTB* meningitis, while the other two patients did not receive any antibiotics prior to CSF testing. In addition, thirteen patients had negative CSF cultures but tested positive on CSF mPCR. Two of these thirteen patients received antibiotics prior to CSF sample collection as seen in Table 2. Pathogens included *Streptococcus pneumoniae* (seven patients), *Haemophilus influenzae* (three patients), *Streptococcus agalactiae* (two patients), and *Escherichia coli* (one patient). All discordant results were confirmed by reviewing the patient’s clinical presentation, CSF analysis, clinical course, and final documented diagnosis.

**Table 1:** Sensitivity, specificity, and OPA between CSF mPCR and CSF culture OPA: overall percentage agreement; CSF: cerebrospinal fluid; mPCR: multiplex polymerase chain reaction

Parameter	Sensitivity	Specificity	OPA
CSF mPCR panel vs. CSF culture	96.3%	100%	99.2%

**Table 2:** CSF samples with discordant results on CSF mPCR panel and CSF culture CSF: cerebrospinal fluid; mPCR: multiplex polymerase chain reaction

Discordant results	Pathogen	Prior antibiotic use
CSF culture positive, mPCR negative (3 cases)	Methicillin-sensitive *Staphylococcus aureus*, *Bacillus cereus*, *Mycobacterium tuberculosis*	*Mycobacterium tuberculosis* case had antibiotics prior to lumbar puncture
CSF culture negative, mPCR positive (13 cases)	*Streptococcus pneumoniae* (7), *Haemophilus influenzae* (3), *Streptococcus agalactiae* (2), *Escherichia coli* (1)	Two of these 13 cases had antibiotics prior to CSF sample collection

The median (interquartile range (IQR)) durations (days) of antimicrobial use in children, adults, and the elderly were 2 (2-7), 6 (2-14), and 7 (2-14), respectively. The total duration of antibiotic use was significantly lower in children (5.6 days) compared to adults (7.3 days) (p=0.005) and the elderly (11.5 days) (p=0.034). The median (IQR) duration (days) of hospitalization in children, adults, and the elderly were 5 (3-10), 6 (3-10), and 9 (4-22), respectively. Children also had a shorter duration of hospitalization (4.6 days) compared to adults (9.8 days) (p=0.001) and elderly (12.3 days) (p=0.018). Differences in length of hospitalization and duration of antibiotic use between adults and the elderly were not statistically significant.

Patients diagnosed with viral meningitis had a shorter duration of hospitalization (5 days vs. 14.1 days, p=0.004) and less antibiotic use (9.5 days vs. 14.8 days, p=0.001) compared to those with bacterial meningitis as seen in Table 3. Of the laboratory parameters, only CSF WBC showed a significant difference, with patients with bacterial meningitis having higher counts compared to those with viral meningitis (median 115 cells/mm^3^ vs. 7 cells/mm^3^, p=0.03) as shown in Table 3. None of the other laboratory parameters, including C-reactive protein, showed any significant difference among patients with viral or bacterial meningitis.

**Table 3:** Duration of hospitalization, duration of antibiotic use, and the CSF WBC count in cases of viral meningitis compared to bacterial meningitis CSF: cerebrospinal fluid

Viral vs. bacterial meningitis	Duration of hospitalization (median, days)	Duration of antibiotic use (median, days)	CSF WBC (median, cells/mm3)
Viral meningitis (n=14) vs. bacterial meningitis (n=16)	5 vs. 14.1 (p = 0.004)	9.5 vs. 14.8 (p = 0.001)	7 vs. 115 (p = 0.03)

## Discussion

CNS infections are critical medical conditions that require prompt attention to prevent negative clinical outcomes. The manifestations of CNS infections can vary widely and may be subtle, particularly in infants and the elderly. This highlights the significance of having a highly sensitive and specific diagnostic tool to ensure timely and accurate diagnosis. While CSF culture has been considered the gold standard for detecting meningitis and encephalitis for many years [6-8], its long turnaround time of 48 hours and susceptibility to prior antibiotic usage can lead to delays in initiating optimal treatment. These delays can result in unnecessary use of empirical antimicrobials, prolonged hospital stays, and increased overall medical costs [9].To address these limitations, novel and rapid molecular techniques have been developed, which can help prevent the unnecessary use of antimicrobials and reduce hospital stays. The utilization of CSF PCR panels has been gaining traction recently; however, there is still limited data on their diagnostic accuracy and potential benefits in managing CNS infections [10]. Therefore, our study aimed to evaluate the performance and potential clinical benefits of the PCR CSF panel in the Middle East, providing valuable insights into managing patients with CNS infections.

In our study, the demographic analysis revealed a slight overrepresentation of males compared to females (55.20% vs 44.80%). These findings are consistent with a study by Seth et al., which demonstrated a male-to-female ratio of 2:1 [11]. Almost half (180, 51%) of our study sample were children, of which 102 (56.7%) were below the age of 12 months. Our findings are consistent with those of Du et al., who reported that among their patients with meningitis, 51.5% were under 12 months of age [10]. These results further support the notion of age-related susceptibility to infections, potentially attributed to the less developed immune system in young children compared to older ones.

While the mPCR panel is highly comprehensive and covers many of the commonly encountered pathogens responsible for CNS infections, it is not an exhaustive panel. For optimal diagnostic performance, the CSF mPCR panel should be coupled with routine CSF cultures, as was highlighted by our study findings. We encountered three patients with negative CSF mPCR that grew MSSA, MTB*,* and *B. cereus* on CSF culture, none of these pathogens are included in the commercially available CSF mPCR panels in routine clinical use. These patients presented with clinical and laboratory findings suggestive of meningitis, necessitating the initiation of antimicrobial treatment while awaiting CSF culture results [8]. Mycobacterial meningitis can have devastating complications if not diagnosed and treated early. CSF should be sent separately for MTB culture and PCR whenever there is a clinical or epidemiological suspicion [9]. This highlights the importance of maintaining a high clinical suspicion and considering diagnostic clues in conjunction with the patient’s clinical presentation to reach an accurate diagnosis and commence early treatment. Similarly, the mPCR panel does not detect *E. coli* capsular types other than K1 [10]. Hence, CSF culture remains necessary whenever CNS infection is suspected in order to detect pathogens not covered by mPCR and to provide information on antibiotic susceptibility [8]. This is consistent with a comparative evaluation of CSF mPCR and CSF culture conducted in a pediatric population in 2016, which reported an overall agreement of 96.20% between the two methods [12].

We diagnosed 13 patients with bacterial meningitis based on positive CSF mPCR results despite negative CSF cultures. Among them, seven tested positive for *S. pneumoniae* on mPCR, three for *H. influenzae*, two for *S. agalactiae*, and one for *E. coli*. Two of these 13 patients received antibiotics in the emergency department prior to CSF sample collection. Among the seven patients with *S. pneumoniae* infection, two had positive gram stains but negative cultures, likely due to antibiotic use in the outpatient setting, prior to presenting to the hospital with symptoms of CNS infection. However, a history of prior antibiotic use is not always reliable in patients presenting with acute symptoms and can be easily missed. Using only conventional CSF culture for diagnosis, CNS infection may have been missed in these patients, leading to inadequate treatment of infection and potentially devastating consequences. These findings emphasize the high sensitivity of CSF mPCR compared to culture, particularly in patients with prior antibiotic exposure. Several studies have shown that the use of oral or systemic antibiotics prior to obtaining CSF samples can result in the normalization of white blood cell count, protein, and glucose, and no growth on CSF culture, leading to delays in diagnosis and treatment of CNS infections [13-15].

The duration of antibiotics use in children was significantly less compared to adults and the elderly, which can be explained by the higher incidence of viral CNS infections in children requiring early discontinuation of antibiotics [9]. Among our patients, the median duration of antimicrobial treatment for children was two days. This is consistent with the study by Lamprini et al., which evaluated the impact of CSF mPCR on the clinical management of children with CNS infections and reported a decrease in the median duration of antibiotics and acyclovir by three days and four days, respectively [9]. Other studies have shown that the routine use of CSF mPCR in clinical practice results in significantly fewer patients receiving antimicrobials overall and a decrease in hospital stay, driven primarily by the prompt diagnosis of enteroviral meningitis, without a significant reduction in the duration of antimicrobial use (except ampicillin) [16-19]. The limitation of our study was the absence of a control group from the pre-CSF mPCR era. Although we examined the duration of antimicrobial treatment and hospitalization, we were unable to compare the results to the pre-CSF mPCR era.

## Conclusions

CSF mPCR is a highly sensitive and specific diagnostic tool that shows great promise in overcoming the limitations of traditional CSF culture, including long turnaround times and susceptibility to prior antibiotic usage. While commercially available CSF mPCR panels are comprehensive, it is important to remember that these panels are not all-inclusive, and our findings emphasize the continued importance of using CSF culture in all patients with suspected CNS infection to capture unusual pathogens. The high cost of mPCR CSF panels is offset by a reduction in healthcare spending in patients with viral CNS infections as a result of a reduction in the length of hospitalization and duration of antibiotic use, as demonstrated in our study. Further research is needed to quantify the wider impact of CSF mPCR panels in reducing antibiotic use and preventing the emergence of antimicrobial resistance in the Middle East.
